# NETQUANT2: automated web-based quantification of neutrophil extracellular traps from fluorescence microscopy

**DOI:** 10.3389/fimmu.2024.1459933

**Published:** 2024-12-04

**Authors:** Johannes Kumra Ahnlide, Louise Thelaus, Fredrik Kahn, Shane van Breda, Pontus Nordenfelt

**Affiliations:** ^1^ Department of Clinical Sciences Lund, Infection Medicine, Faculty of Medicine, Lund University, Lund, Sweden; ^2^ Department of Infection Medicine, Skåne University Hospital, Lund, Sweden; ^3^ Department of Biomedicine, Laboratory for Experimental Rheumatology, University Hospital Basel, Basel, Switzerland; ^4^ Department of Laboratory Medicine, Clinical Microbiology, Skåne University Hospital, Lund, Sweden

**Keywords:** neutrophil extracellular traps, NEtosis, automated image analysis, neutrophils, fluorescence microscopy

## Abstract

Neutrophil extracellular traps (NETs) are structures that neutrophils form in response to various stimuli, including invading pathogens. NETs are increasingly studied, and their importance has been demonstrated in autoimmunity and infection. However, no consensus has emerged on their quantification, with many studies resorting to manually counting NETs in microscopy images. NETQUANT is a free software for the automated quantification of neutrophil extracellular traps in fluorescence microscopy images. By employing automated image analysis based on biologically relevant criteria for defining NETs, NETQUANT eliminates user bias and reduces analysis time. Despite these advantages, NETQUANT has not reached widespread adoption, partly due to its dependence on proprietary software and challenges associated with local program setup, which has hindered its appeal. Here, we present NETQUANT2, an improved version based on the principles of NETQUANT, released as a web-based software for fast, simple, and unbiased NET quantification from microscopy images. The software guides researchers by displaying relevant morphological data from their sample and allows researchers to interactively configure the analysis, immediately seeing the impact on the result. NETQUANT2 further improves NETQUANT by enabling easy sharing and reusing of configurations and results and enhanced configuration options to handle complex samples better. We believe that the improved accessibility of NETQUANT2 will lead to better reproducibility in NET research and open the field to more researchers while keeping the quality of analysis high.

## Introduction

Neutrophils, the most abundant white blood cells in humans, are crucial in combating infections through various defense mechanisms. The described behaviors that neutrophils exhibit when encountering pathogens include phagocytosis, degranulation, and the formation of neutrophil extracellular traps (NETs) (1). Of these behaviors, the formation of NETs is the most recently identified (2). Since its initial description as a form of cell death with antimicrobial effects, the description of NETs has grown more complex. Neutrophils form NETs by expelling modified chromatin into the extracellular space, but this is now known to happen through various pathways, not all of which lead to cell lysis (3). NET formation appears to be evolutionarily conserved, highlighting its importance in host defense (4). However, NETs are also implicated in the pathogenesis of several immune and infectious diseases, including systemic lupus erythematosus, chronic obstructive pulmonary disease, and severe COVID-19 (5–7).

There is a growing interest in NETs, and many of the studies are performed *in vitro* using some method of NET quantification (8). Despite this widespread need for quantitative NET analysis, no consensus has emerged on quantification, and many studies resort to manually counting NETs in microscopy images. Among the ways used to quantify NETosis are enzyme-linked immunosorbent assay (ELISA) targeting complexes of DNA and proteins associated with NETosis such as neutrophil elastase (NE) and myeloperoxidase (MPO) (6, 9) and flow cytometry-based quantification measuring cells with extracellular DNA (10, 11). However, a microscopy-based method is needed to observe and quantify NETs directly.

Different staining methods can be used to visualize NETs in microscopy (12). DNA-intercalating dyes such as DAPI, SYTOX Green, or SYTOX Orange are often used to stain the chromatin in NETs. Since SYTOX Green and SYTOX Orange are cell-impermeable, they do not stain intracellular DNA, and some methods rely entirely on the presence of this extracellular DNA as a signal for NETosis (13). However, unless such a method is complemented by another technique or study of DNA morphology, it is impossible to distinguish NET formation from other causes of extracellular DNA, such as necrosis. Immunostaining is frequently used to increase specificity for NETs. Common targets include NE and MPO, which promote chromatin decondensation during NET formation and are subsequently found in the NET (2, 14). Another possibility is staining with antibodies against DNA and histone complexes, as histones are the most abundant protein in NETs (15, 16). If the goal is assessment based on cell count, e.g. quantification of the proportion of cells that form NETs, then a common and experimentally easy approach is to use DAPI that stains the DNA of neutrophils and at least one immunofluorescent stain specific for NETs.

The microscopy images need to be analyzed and interpreted to quantify NET formation. A human can perform this task, but this is time-consuming and introduces individual bias. To eliminate individual bias and enable high-content screening, researchers have been looking for automated image analysis methods to perform this task (8, 17, 18). NETQUANT introduced an algorithm for automated NET quantification based on three biologically relevant morphological criteria (19). The algorithm relies on two stains, a DNA stain and a stain of a protein inside neutrophils that localize to the NET, e.g. DAPI and NE. As criteria suggestive of NETosis, NETQUANT uses the increase in the area of the NET-associated protein, increase in DNA area relative to NET-associated protein, and increased deformation of DNA. While the increase in DNA area relative to the area of the cell is seen, e.g., in the early stages of NETosis, NETQUANT focuses on quantifying NETs. Cells with an intact cell membrane in the earlier phases of NETosis (intact NETotic), where chromatin decondensation and destruction of the nuclear envelope has started, are not targeted. The algorithm was distributed as a free software written in MATLAB that, once set up, could automatically analyze microscopy images and output the percentage of NET formation. However, despite being recognized as a valuable tool (18), the need for a proprietary language and knowledge in programming and the absence of tools that help the user set the algorithm parameters have hindered its widespread use (8, 17, 18).

Here, we introduce NETQUANT2, a web-based software that uses and expands on the biologically relevant criteria in NETQUANT while designed to address accessibility and usability barriers. This next-generation tool shifts from a proprietary environment to an easy-to-use web-based platform, facilitating broader adoption without requiring specialized software knowledge. Moreover, the software incorporates features like manual annotation, automatic settings for thresholding, and generation of visualizations and data suitable for advanced analyses. By streamlining the workflow and ensuring high adaptability, NETQUANT2 aims to standardize NET quantification across varied research contexts.

## Results

### Overview of NETQUANT2

**Figure 1:**
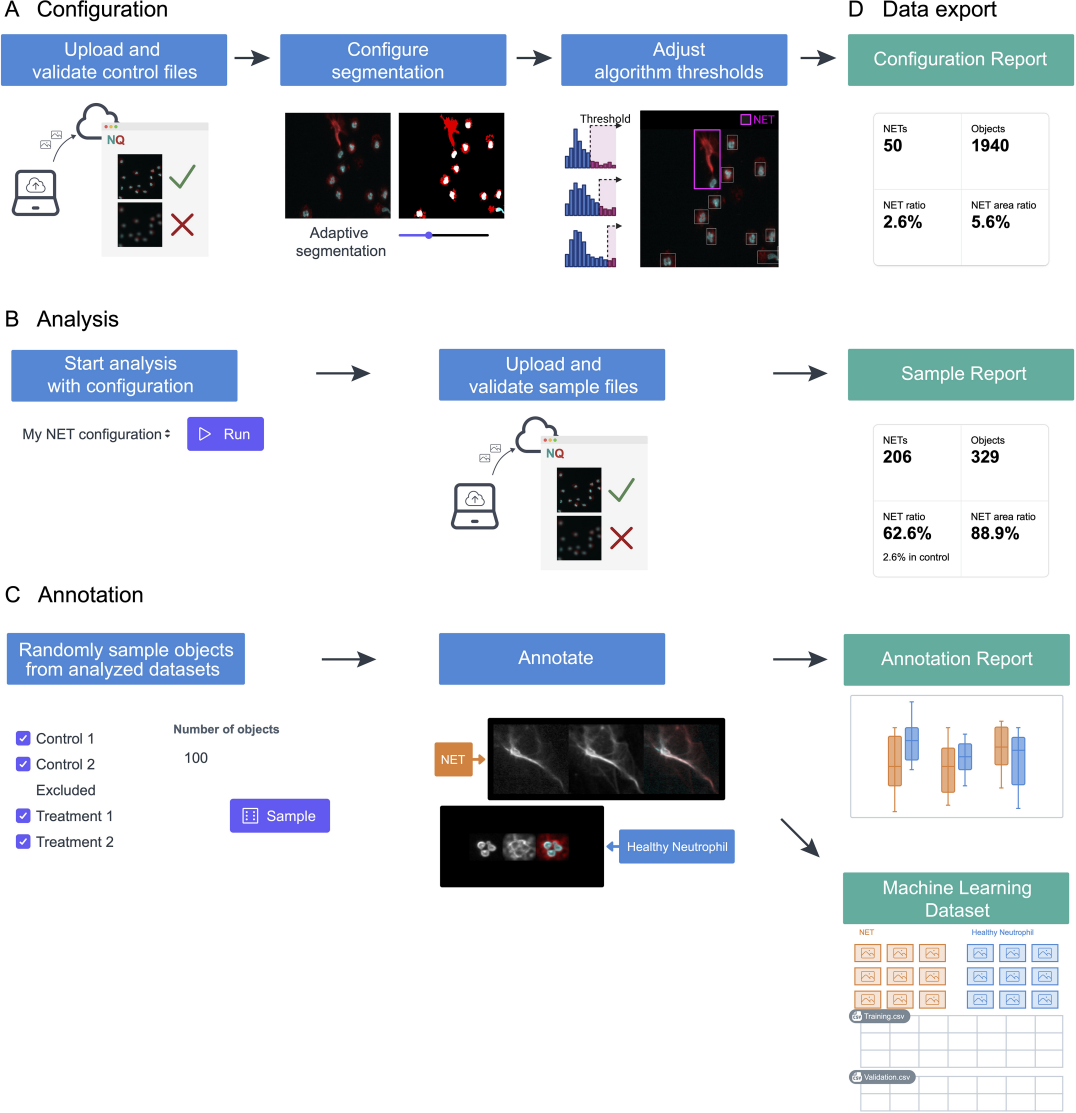
Schematic representation of the NETosis analysis workflow using NETQUANT2. **(A)** To build a configuration, files from a negative control dataset are uploaded to the web interface where fields of view that have quality issues can be excluded. The image segmentation parameters and algorithm thresholds are adjusted interactively using this dataset. **(B)** The configuration can then be used to run the analysis with the predefined settings for multiple datasets. **(C)** NETQUANT2 additionally contains a tool for manual annotation to validate and inform the automated analysis. Using the tool users can randomly sample objects and easily create an annotated dataset. **(D)** Each step produces relevant visualizations and summary statistics as well as CSV files that can be used for further analysis. The annotation report further includes automated adjustment of algorithm thresholds and the annotations can be exported for use in machine learning.

The NETQUANT2 software streamlines the NETosis analysis through an efficient and user-friendly web-based workflow, as depicted in **Figure 1**. Control datasets are uploaded to the platform and used to interactively configure segmentation and adjust algorithm thresholds stored on the server (**Figure 1A**). This setup enables the application of predefined settings across multiple datasets, ensuring consistency and reproducibility (**Figure 1B**). While the application is not intended for long-term storage of images, all fields-of-view can be viewed for the duration of configuration and analysis with interactive analysis results overlaid on the images providing the context for the result and aiding interpretation. Moreover, NETQUANT2 incorporates a manual annotation tool that enhances the validation process, enabling users to randomly sample and annotate objects to build a robust dataset that informs the automated analysis without introducing hidden parameters (**Figure 1C**). Each step of the workflow generates outputs, including visualizations and summary statistics, alongside CSV files (**Figure 1D**). These files support further research and machine learning applications. This integrated approach simplifies the analysis and ensures that it is comprehensive and adaptable to varied research needs.

### Direct two-dimensional projection can cluster phenotypic variation in NET samples

In any sample, there will be significant heterogeneity. Prior published knowledge and experience from microscopy about behaviors and their appearance in the microscope enables the creation of specific relevant features to account for some of this heterogeneity. These features impose a structure on the data that enables the creation of explainable, unbiased quantification algorithms. The morphological measurements that NETQUANT and NETQUANT2 build on are easy to understand and correspond to changes in morphology expected in NETosis. This should make them robust to variation between samples, provided the central assumptions about NET formation still hold. However, any selection of features offers a limited view of the data, which may obscure important information. Objects may exhibit unexpected phenotypes, artefacts, or differences in the appearance of behaviors. This evaluation is often made by the microscopist acquiring the images before quantification, but since the features used in NETQUANT2 are derived from microscopy images of the individual objects, unsupervised machine learning methods can be used to make a more complete unbiased assessment of heterogeneity.

**Figure 2:**
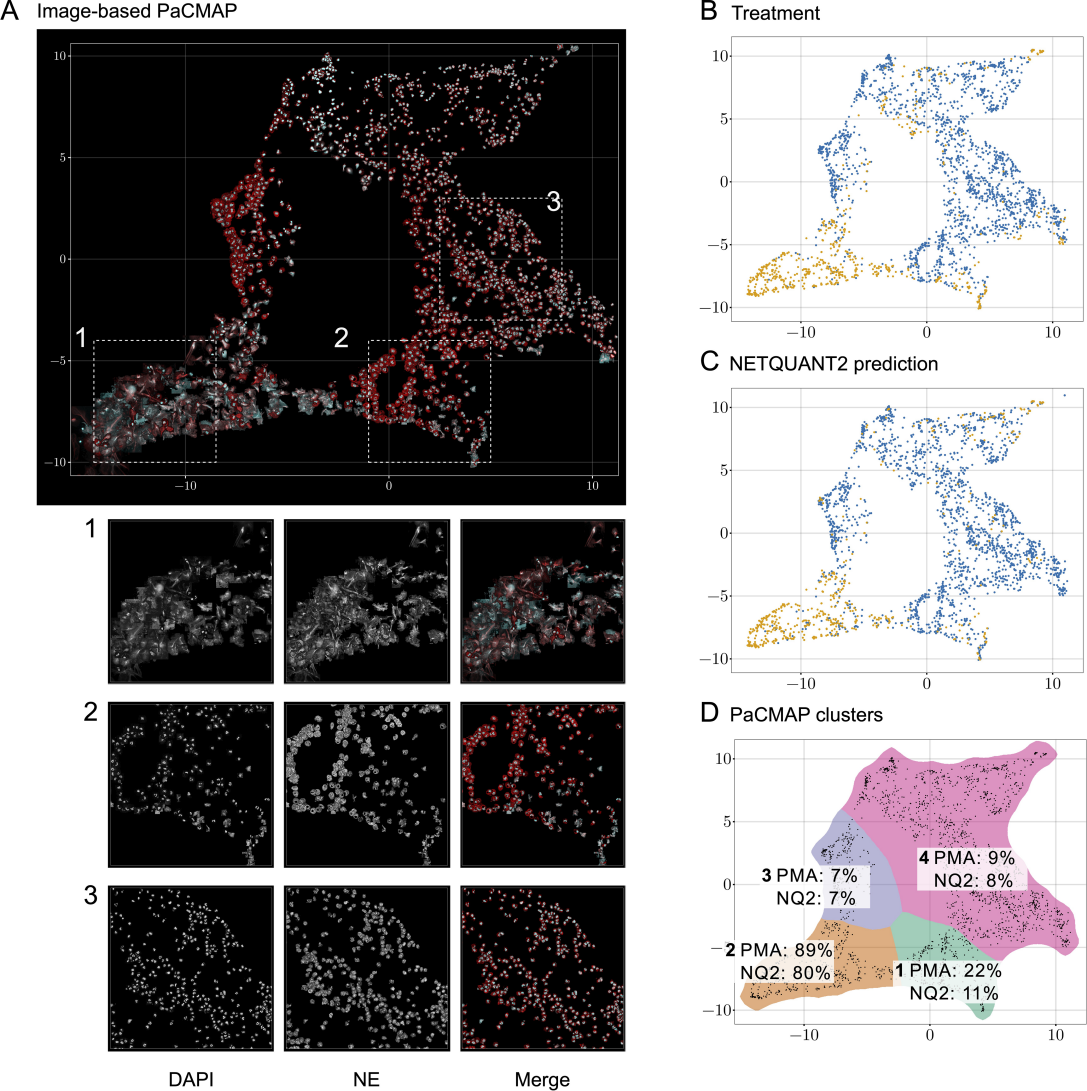
Direct two-dimensional projection can cluster phenotypic variation in NET samples. **(A)** Dimensionality reduction using PaCMAP on fluorescence signals reveals multiple distinct phenotypes present in neutrophil samples. Images of objects in the neutrophil samples plotted at their position in a PaCMAP of the radial projection of their fluorescence microscopy signal (3 donors, 2190 cells). Clusters corresponding to different phenotypes are seen in different parts of the plot. Three different areas are picked out as examples and shown in zoomed-in views below. For these areas, the DNA channel (left) and neutrophil elastase channel (middle) as well as a merge are shown. **(B)** PMA-treated cells mainly cluster to one region of the PaCMAP. Each dot in the scatter plot corresponds to an individual detected object, colored by treatment condition—control in blue and PMA in orange—showing the distinct distribution patterns associated with each treatment. **(C)** Algorithmic prediction of NET formation. A similar scatter plot classifies cells based on NETQUANT2 predictions, with ‘NET’ in blue and ‘Not NET’ in yellow. While mostly targeting the PMA cluster the algorithm also classifies objects from certain other clusters as NETs. **(D)** PMA-stimulated cell proportion and proportion of cells predicted to be NETs in PaCMAP clusters. Taking the data in **(B, C)** together with a manual selection of apparent clusters quantifies the observed patterns. In the mostly clearly NETotic cluster 2 a large proportion of cells come from the PMA-stimulated samples and a large proportion of cells are classified as NETs by the NETQUANT2 algorithm. All other clusters mostly contain cells from the control sample. In cluster 1 and 3 the cells seem fairly large and have a high signal in the NE channel, possibly indicating some kind of activation. In both of these clusters there are also some smaller NET-like structures. These structures could be fragments of NETs or smaller NETs released through vital NETosis. Cluster 4 contains mostly healthy neutrophils that are smaller than the ones in cluster 1 and 3.

To analyze the heterogeneity of objects in the NETQUANT dataset, a two-dimensional data projection of the fluorescence signal was used, which revealed phenotypic variation partly due to NETosis. The dataset used for the original NETQUANT paper, neutrophils from healthy donors untreated or stimulated with phorbol 12-myristate 13-acetate (PMA) to promote NETosis, was analyzed using NETQUANT2. To expose structure in the staining patterns of the neutrophil population without presupposing phenotypes (e.g., NETs and neutrophils), dimensionality reduction (DR) was performed on the objects found by NETQUANT2. Instead of using the raw image data for DR, the pixels were binned by distance to the object centroid. The mean of each bin was taken to ensure that the signal was invariant to rotation in the original image. By projecting this radial fluorescence signal in the neutrophil elastase and DNA channel on a two-dimensional space using pairwise controlled manifold approximation (PaCMAP) (20), distinct phenotypes were mapped and could be visually differentiated (**Figures 2A, D**). The projection suggests a few different clusters with PMA-treated cells to a significant degree clustering in one region (**Figure 2B**). Plotting the NETQUANT2 algorithm prediction in this space shows that the algorithm mainly predicts cells in the PMA cluster as NETs, while also classifying some cells in an adjacent cluster as NETs (**Figure 2C**). This suggests that the biologically relevant criteria used in the algorithm, to a large degree, explain the phenotypic variation introduced by PMA stimulation.

The heterogeneity analysis above shows the large variation in NE and DNA signal between neutrophils from healthy donors and suggests further phenotypes exist beyond those targeted here and by the original NETQUANT software. Possible sources of variation beyond complete NETosis include neutrophil activation, exocytosis, early stages of NET formation, and neutrophils migrating after NET formation. This highlights the importance of unbiased analysis allowing the researcher to find the phenotypes in their samples and to make active decisions about what to quantify.

### New NET criteria and automatic thresholding improve NET identification

NETQUANT2 introduces a new manual validation functionality that allows users to randomly sample from the objects identified and add structured annotations to them using a simple interface. After choosing the number of objects to annotate and using the sampling strategy, users are shown the images randomly and asked to pick a label to assign to each image. Here the labels “NET”, “Intact NETotic”, “Multiple intact cells”, “Neutrophil”, “Not a cell”, and “Unknown” were chosen. The annotations can be exported and used in further analysis, e.g., for machine learning, and the built-in visualization and analysis pages enable manual and automatic configuration of the thresholds based on the data.

**Figure 3:**
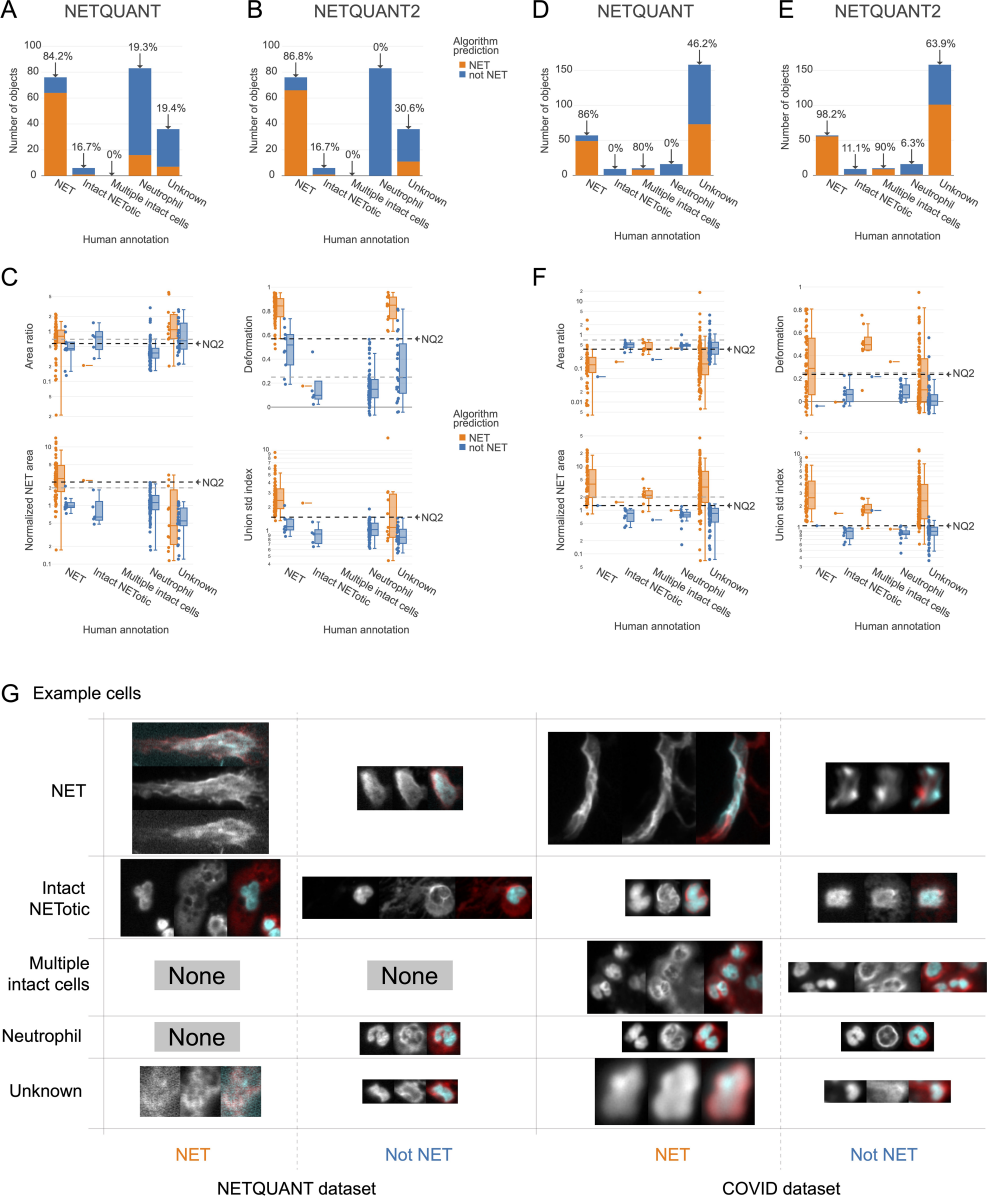
New NET criteria and automatic thresholding improve NET identification. Using the annotation functionality and the new algorithm a high level of agreement between prediction and annotation can be achieved. **(A, B)** Comparison of human annotation and algorithm outputs for NETQUANT with default parameters **(A)** and the proposed algorithm, NETQUANT2 with thresholds set automatically from the Neutrophil and NET groups in the human annotations **(B)**. Percentages indicate the proportion of objects identified as NETs by the algorithm in each group. **(C)** Box plot of features of the objects grouped by human annotation with subgroups for positive or negative NET prediction according to the NETQUANT2 algorithm with thresholds as in **(B)**. The used threshold levels are indicated by a black dashed line marked NQ2. Default values of features in the original NETQUANT algorithm are indicated by gray dashed lines. **(D–F)** The extension of the validation process to a COVID-19 patient sputum dataset reveals heightened complexity, as a significant number of objects are marked ‘Unknown’ by human annotators. These plots mirror the analyses in **(A–C)**. **(G)** Fluorescence microscopy images from the NETQUANT and COVID datasets for different human annotations and NETQUANT2 algorithm prediction.

A high level of agreement between prediction and human annotation could be achieved using the manual validation functionality and the new algorithm. A manual annotation was performed with 250 objects from the two datasets. The two datasets chosen were the images acquired for the original NETQUANT article of neutrophils from healthy donors and sputum samples from patients with severe COVID-19 from a previous study (21). For the objects labeled as NETs or neutrophils in the manual annotation, the original NETQUANT algorithm agreed with the annotation in 82% of the cases and out of the objects that the algorithm predicted to be NETs, it was 20% (**Figure 3A**). By using the new algorithm, which includes the standard deviation of the radius as a criterion and the functionality in the NETQUANT2 software to automatically set thresholds from an annotation, an accuracy of 95% and an FDR of 0% could be achieved (**Figures 3B, C**). Repeating the process for the second, more difficult, dataset gave an accuracy of 89% and an FDR of 0% (**Figures 3D**) for the original NETQUANT algorithm with NETQUANT2 achieving an accuracy of 97% with an FDR of 3% (**Figures 3E, F**). Some differences in NET characteristics between the datasets are apparent, e.g. with regard to the deformation (**Figures 3C, F**). Both in the new version and the old version, clusters of multiple cells with intact nuclei tend to be classified as NETs. In contrast, single cells that appear intact but swollen or starting to produce NETs are generally classified by the algorithm as not NETs. It should be noted that it can be difficult even for an expert to label all objects correctly. Importantly, the automatic adjustments made to the parameters are not hidden and can be interpreted by the researcher in relation to their experiment. The example images showcase the complexity faced by both annotator and algorithm, including low signal, out-of-focus structures, and embedding of cells in larger structures (**Figure 3G**). The introduction of the new feature and the inclusion of tools to perform and take full advantage of manual annotation empowers biologists to make informed decisions and reach reproducible automated NET quantification based on biologically relevant morphological features.

### Web-based user interface enables easy access and efficient NET analysis

**Figure 4:**
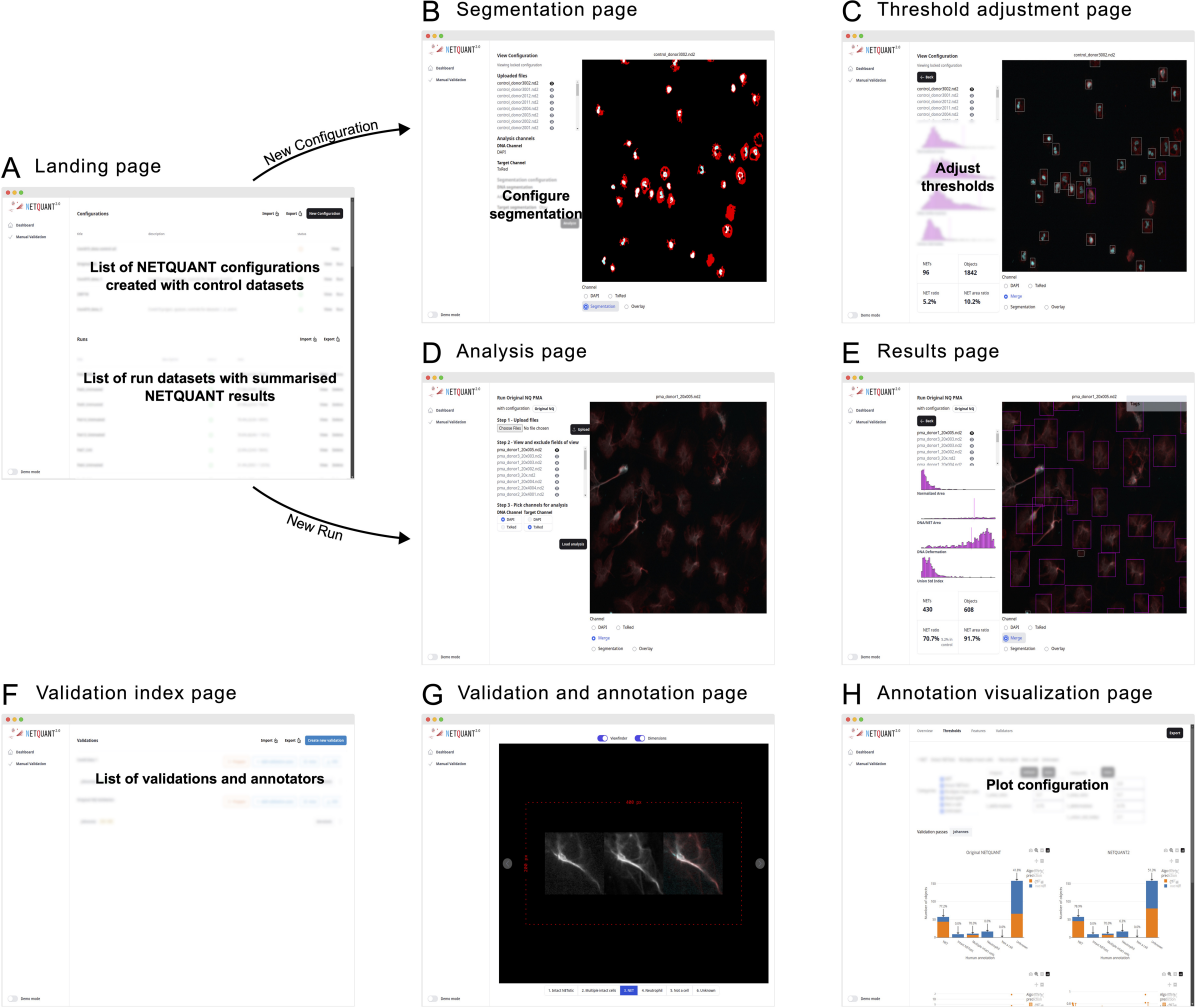
Web-based user interface enables easy access and efficient NET analysis. **(A)** The NETQUANT2 landing page shows users a summary of their configuration and analyzed datasets. On the page, they can manage and create configurations, initiate new dataset analyses, or review results. The lists of configurations and analyzed datasets can be exported to a CSV file and shared with other users. **(B)** When creating a new configuration users upload a control dataset on which they interactively configure the parameters of the analysis. The user picks and configures a segmentation algorithm for each channel and the resulting binary masks used for segmentation are shown to the right. **(C)** After running the initial analysis, NETQUANT2 algorithm thresholds can be adjusted to ensure that the objects in the sample are correctly identified. The objects are shown in an interactive overlay on the image and colored according to the prediction made by the algorithm. These objects can be selected to view the position of their features in the overall distribution which reveals the reason for the algorithm prediction to the user. **(D)** Minimal user input is needed to run a new sample with a previous configuration. If desired users can interactively exclude files or fields of view that are not appropriate for analysis. Once the files are uploaded and the appropriate channels selected, the pre-configured analysis is run with the click of a button. **(E)** The analysis results in an interactive sample report. An interactive overlay like in **(C)** allows users to explore the identified objects and their feature distribution. The report contains summary statistics including the number of objects, the number and percentage of NETs, and the percentage of object area covered by NETs. **(F)** The manual validation index page gives users an overview of validations performed using NETQUANT2’s built-in annotation and validation tool. New validations can be created by configuring how objects should be sampled from multiple datasets. Every such random sample can be annotated multiple times by different people. **(G, H)** The new annotation and validation tool simplifies manual quality control, algorithm interpretation, and algorithm configuration. Through the annotation interface **(G)** annotators assign a label to every object in the sample. These annotations are aggregated and visualized in various ways providing users with an overview of algorithm performance and allowing for automatic adjustment of algorithm thresholds.

The NETQUANT2 software is provided through a web-based user interface designed to be easy to use and highly interactive (**Figure 4**). Upon accessing the landing page, users are presented with options to manage configurations, run analyses using the configurations, and review results. This workspace can be exported in CSV format and shared with collaborators, who can work on the same runs and configurations.

Creating a new configuration is interactive; users upload control datasets, select segmentation algorithms, and adjust algorithm thresholds to refine object identification, supported by visual feedback through interactive overlays (**Figure 4**). On the landing page (**Figure 4A**) users can create new configurations giving them a name and a description. They are then guided through uploading their images and visualizing them in the web interface. In this step, bad fields-of-view can be excluded from further analysis. After uploading the images and picking which channels in the images correspond to the DNA stain and the NET associated protein stain the users configure the segmentation of these channels (**Figure 4B**). When adjusting the segmentation parameters, a visual of the resulting mask is shown overlaid on the image. Once the users have set up the segmentation so that it correctly segments each channel the analysis can be run for all images. Running the analysis shows the thresholds adjustment page (**Figure 4C**). Colored bounding boxes are overlaid on the images showing every identified object and indicating if the algorithm predicts that an object is a NET. The users can interactively adjust the parameters of the algorithm and see the bounding boxes change color based on the updated prediction. The users save these configurations based on control datasets after which they become available on the landing page to use for analysis.

When users run an analysis using a configuration no adjustment of segmentation or thresholds is made. The images, acquired using the same imaging protocol as the control dataset used for the configuration, are uploaded to the analysis page (**Figure 4D**). On this page the users can view the images and exclude bad images after which they can apply the analysis and view the results (**Figures 4E, F**) including how the samples were segmented, and the prediction for every object overlaid on the images.

The manual validation tool supports quality control by allowing multiple annotators to label objects (**Figure 4G**). The resulting annotated data is presented in interactive plots where users can tweak the algorithm thresholds and see the results of the algorithm’s prediction for the different labels. The performance of the algorithm with the selected thresholds is shown in various ways (**Figure 4H**) and users can use the auto threshold functionality to find thresholds that separate different sets of labels in terms of the NET prediction.

## Discussion

By requiring minimal setup on the part of the researcher, NETQUANT2 puts fully automated single-cell image-based analysis in every NET researcher’s toolbox. If the research question is not about what happens to neutrophils but rather how they affect their environment, alternative methods of automatic quantification are available (12, 18). In such cases, distinguishing between NETosis and necrosis might not be crucial. Instead, the total amount of released extracellular DNA could be of interest. When the specifics of NET formation are important, observations of NETs are made at the single object level (12). Recent increases in the number of studies using hand counting of NETs as the quantification method indicate that this interest in direct observation is growing, and researchers would benefit from better, easy-to-use tools (8). The inherent complexity of single-cell NET quantification can be brought to the forefront by removing the incidental complexity of software setup and managing annotations.

While standardization of image analysis through NETQUANT2 is an important step towards making results in the field comparable and reproducible, other sources of variability remain. Different studies use different protocols to generate NETs, including different methods of stimulation, in addition to the differences in visualization and quantification. In this study, both a dataset using PMA-stimulation and clinical samples without additional stimulation were used to benchmark the method. NETQUANT2 was applied successfully to both datasets, adjusting the parameters to account for the differences in the datasets. However, the results of future studies will only be comparable when the same or similar protocols are used. When the same protocol is used, the same configuration of the algorithm can be used, further eliminating sources of bias and variability. Although this study does not directly address the need for the field to converge on a few standardized protocols, we believe that the easy sharing of configuration and results enabled by NETQUANT2 will help push the field in this direction.

As shown here and elsewhere, there is a large phenotypic variability in neutrophil NET production, which causes problems not only for automated methods but also for researchers (8). While the research community is still figuring out the fundamentals of NET formation, subjectivity in labeling will not just be due to personal bias but also to explicit or implicit choices made by researchers. Researchers could be interested in the early stages of NET formation and include any cell that shows signs of a decondensed nucleus (22). Furthermore, it is not immediately obvious how a cluster of neutrophils embedded in a NET should be classified or how to count viable neutrophils that have produced NETs and continued migrating. While NETQUANT2 makes automatic quantification easy, researchers still need to make active decisions about the definition of NETs and validate their assumptions through the interactive tools available in the software. For the purposes of this study, NETs created through vital NETosis, by a neutrophil that subsequently continued migrating, were treated the same as lytic NETs. By annotating a dataset specific to lytic NETs, authors can automatically or manually fit the algorithm parameters so that other NETs are excluded to the extent possible. While we have not tested such an exclusion in this study, the algorithm could likely be made more specific to lytic NETs, e.g. by adjusting the parameters for increased emphasis on the area of the NET-associated protein. The ambiguity in the definition of NETs is inherent to the study of NETosis and further highlights the importance of an explainable approach to quantification. The integrated annotation and visualization tooling in NETQUANT2 connects the subjective choices of annotators back to biologically relevant features.

More research and development are needed to cover the need for image-based quantification in the NETosis field. In this study, we have shown how NETQUANT2 can be used to classify objects as NETs or not NETs in a population of neutrophils. However, more complicated scenarios are also of interest to researchers. In cases where the different stages or different types of NETosis need to be quantified, a more detailed classification is necessary. The same is true for quantifying NET formation in tissues with multiple cell types with more variable morphologies. In the future, it could be interesting to investigate if NETQUANT2 could inform or be combined with other cell classification algorithms to cover this gap.

Machine learning approaches could conceivably automatically make the judgments currently made by humans (23). Including annotation tools and export functionality for downstream machine learning in NETQUANT2 promotes further exploration of this avenue and collection of datasets that could be valuable in the future. However, going down the route of supervised machine learning too quickly risks implicitly encoding the assumptions of a few researchers in an opaque, hard-to-reason model. To capture all the complexities of NETs, more researchers need to do manual annotations in a standardized way and share the results with each other.

## Method

### Data acquisition

The datasets used were acquired in previous studies (19, 21), and the reader is referred to these studies for a full description of the experimental setup. In brief, for the NETQUANT dataset, neutrophils were isolated from blood collected in heparin tubes using a two-step sedimentation and centrifugation protocol with 2% Dextran in 0.9% NaCl. Erythrocytes were lysed, and neutrophils were resuspended in RPMI-1640 medium containing 2 mg/ml HSA. Neutrophils were seeded on poly-l-lysine-coated coverslips and stimulated to form neutrophil extracellular traps (NETs) using 20 nM PMA for 150 min. After stimulation, cells were fixed with 4% paraformaldehyde and stained for DNA and neutrophil elastase using DAPI and Alexa594-labeled antibodies, respectively. Fluorescence microscopy images were acquired with NIS-elements 5.1 using a Nikon Ti-E microscope equipped with either an Andor Zyla 4.2 or a Hamamatsu Orca CCD camera with a Plan Apochromat 20× objective. For the sputum samples in the COVID-19 dataset, sputum was collected from COVID-19 patients and healthy controls and immediately fixed with 4% paraformaldehyde in PBS at 4°C for 1 h. The fixed sputum was diluted in PBS and cytocentrifuged onto glass slides. After permeabilization and blocking, samples were stained with rabbit anti-human neutrophil elastase antiserum and Alexa Fluor (AF)-647–conjugated goat anti-rabbit Fab2’ antibody fragments, while DNA was stained with DAPI. Widefield fluorescence microscopy images were acquired with NIS Elements AR using a Nikon Ti-2 inverted microscope equipped with a 20×/0.75 objective and a Nikon DS-Qi2 sCMOS camera. Fluorophores were excited with a Lumencor SpectraX light engine, and emission was collected with appropriate dichroic mirrors and filters.

### Image analysis

The image analysis functionality of NETQUANT2 is written in Julia (24) and was for the analyses in this study run on version 1.10.2. Multiple options for image binarization are available as part of the segmentation step. Two of these options were used for the analyses in this study. The first option was adaptive thresholding (25), which is used with a window size of 1/8 of the average of the width and height of the image rounded to the nearest integer and a modification of the algorithm that allows for negative percentages for the threshold. The second option used was Otsu’s method (26).

For the NETQUANT dataset, the DNA channel was binarized using adaptive thresholding with -18% for the threshold, and the neutrophil elastase channel was binarized using Otsu’s method. For the COVID-19 dataset, the DNA channel was binarized using Otsu’s method, and the neutrophil elastase channel was binarized using adaptive thresholding with -5% for the threshold. If multiple DNA segments were in the same neutrophil elastase segment and more than 5 pixels apart, watershed segmentation was applied to separate them.

### Datasets and statistics

The NETQUANT dataset contains images of neutrophils isolated from 3 healthy donors. For the control condition, there are a total of 30 fields-of-view, excluding clearly overlapping fields-of-view. The dataset contains 19 images of the PMA-stimulated condition, excluding clear duplicates. In this study, 250 objects randomly sampled from the whole dataset were annotated by one annotator. Similarly, 250 objects were annotated by one annotator from the COVID dataset of 158 images from 3 COVID patients and 1 healthy donor. Based on this annotation the objects that were marked with the “NET” label were considered true positives (TP) if predicted to be NETs by the algorithm and false negatives (FN) otherwise. Objects marked “neutrophil” were considered false positives if predicted to be NETs and true negatives (TN) otherwise. Based on this, accuracy and FDR were calculated according to standard definitions:


$$ACC=\frac{TP+TN}{TP+FN+TN+FP},\;FDR=\frac{FP}{TP+FP}$$

### Software availability

NETQUANT2 is currently hosted at https://netquant.bmc.lu.se/. The user manual can be accessed by clicking the “Demo mode” toggle in the left-hand corner. For long-term support and software updates, please see https://www.nordenfeltlab.com/software/.

## Data Availability

The raw data supporting the conclusions of this article will be made available by the authors, without undue reservation.

## References

[1] Ley K Hoffman HM Kubes P Cassatella MA Zychlinsky A Hedrick CC . Neutrophils: New insights and open questions. Sci Immunol. (2018) 3:eaat4579. doi: 10.1126/sciimmunol.aat4579 30530726

[2] Brinkmann V Reichard U Goosmann C Fauler B Uhlemann Y Weiss DS . Neutrophil extracellular traps kill bacteria. Science. (2004) 303:1532–5. doi: 10.1126/science.1092385 15001782

[3] Yipp BG Petri B Salina D Jenne CN Scott BNV Zbytnuik LD . Infection-induced NETosis is a dynamic process involving neutrophil multitasking in *vivo* . Nat Med. (2012) 18:1386–93. doi: 10.1038/nm.2847 PMC4529131 22922410

[4] Robb CT Dyrynda EA Gray RD Rossi AG Smith VJ . Invertebrate extracellular phagocyte traps show that chromatin is an ancient defence weapon. Nat Commun. (2014) 5:4627. doi: 10.1038/ncomms5627 25115909 PMC4143918

[5] Garcia-Romo GS Caielli S Vega B Connolly J Allantaz F Xu Z . Netting neutrophils are major inducers of type I IFN production in pediatric systemic lupus erythematosus. Sci Transl Med. (2011) 3:73ra20–0. doi: 10.1126/scitranslmed.3001201 PMC3143837 21389264

[6] Dicker AJ Crichton ML Pumphrey EG Cassidy AJ Suarez-Cuartin G Sibila O . Neutrophil extracellular traps are associated with disease severity and microbiota diversity in patients with chronic obstructive pulmonary disease. J Allergy Clin Immunol. (2018) 141:117–27. doi: 10.1016/j.jaci.2017.04.022 PMC5751731 28506850

[7] Middleton EA He X-Y Denorme F Campbell RA Ng D Salvatore SP . Neutrophil extracellular traps contribute to immunothrombosis in COVID-19 acute respiratory distress syndrome. Blood. (2020) 136:1169–79. doi: 10.1182/blood.2020007008 PMC7472714 32597954

[8] Henneck T Krüger C Nerlich A Langer M Fingerhut L Bonilla MC . Comparison of NET quantification methods based on immunofluorescence microscopy: Hand-counting, semi-automated and automated evaluations. Heliyon. (2023) 9:e16982. doi: 10.1016/j.heliyon.2023.e16982 37484269 PMC10361044

[9] Kessenbrock K Schönermarck U Back W Gross WL Werb Z Gröne H-J . Netting neutrophils in autoimmune small-vessel vasculitis. Nat Med. (2009) 15:623–5. doi: 10.1038/nm.1959 PMC2760083 19448636

[10] Gavillet M Renella R Harris C Shapiro NI Wagner DD Williams DA . Flow cytometric assay for direct quantification of neutrophil extracellular traps in blood samples. Am J Hematol. (2015) 90:1155–8. doi: 10.1002/ajh.v90.12 PMC4715743 26347989

[11] Zharkova O Tay SH Lee HY Shubhita T Ong WY Lateef A . A flow cytometry-based assay for high-throughput detection and quantification of neutrophil extracellular traps in mixed cell populations. Cytometry A. (2019) 95:268–78. doi: 10.1002/cyto.a.23672 PMC6590256 30549398

[12] de Buhr N von Köckritz-Blickwede M . How neutrophil extracellular traps become visible. J Immunol Res. (2016) 2016:4604713. doi: 10.1155/2016/4604713 27294157 PMC4884809

[13] Carmona-Rivera C Kaplan MJ . Induction and quantification of NETosis. Curr Protoc Immunol. (2016) 115:14.41.1–14.41.14. doi: 10.1002/0471142735.2016.115.issue-1 27801512

[14] Papayannopoulos V Metzler KD Hakkim A Zychlinsky A . Neutrophil elastase and myeloperoxidase regulate the formation of neutrophil extracellular traps. J Cell Biol. (2010) 191:677–91. doi: 10.1083/jcb.201006052 PMC3003309 20974816

[15] Urban CF Ermert D Schmid M Abu-Abed U Goosmann C Nacken W . Neutrophil Extracellular Traps Contain Calprotectin, a Cytosolic Protein Complex Involved in Host Defense against Candida albicans. PloS Pathog. (2009) 5:e1000639. doi: 10.1371/journal.ppat.1000639 19876394 PMC2763347

[16] Neumann A Völlger L Berends ETM Molhoek EM Stapels DAC Midon M . Novel role of the antimicrobial peptide LL-37 in the protection of neutrophil extracellular traps against degradation by bacterial nucleases. J Innate Immun. (2014) 6:860–8. doi: 10.1159/000363699 PMC4201878 25012862

[17] Brinkmann V Goosmann C Kühn L Zychlinsky A . Automatic quantification of in *vitro* NET formation. Front Immunol. (2013) 3. doi: 10.3389/fimmu.2012.00413 PMC3540390 23316198

[18] van Breda SV Vokalova L Neugebauer C Rossi SW Hahn S Hasler P . Computational Methodologies for the in *vitro* and in *situ* Quantification of Neutrophil Extracellular Traps. Front Immunol. (2019) 10:1562. doi: 10.3389/fimmu.2019.01562 31354718 PMC6635468

[19] Mohanty T Sørensen OE Nordenfelt P . NETQUANT: automated quantification of neutrophil extracellular traps. Front Immunol. (2018) 8. doi: 10.3389/fimmu.2017.01999 PMC5775513 29379509

[20] Wang Y Huang H Rudin C Shaposhnik Y . Understanding how dimension reduction tools work: an empirical approach to deciphering t-SNE, UMAP, triMap, and paCMAP for data visualization. J Mach Learn Res. (2021) 22:1–73. doi: 10.5555/3546258.3546459

[21] Fisher J Mohanty T Karlsson CAQ Khademi SMH Malmström E Frigyesi A . Proteome profiling of recombinant DNase therapy in reducing NETs and aiding recovery in COVID-19 patients. Mol Cell Proteomics. (2021) 20:100113. doi: 10.1016/j.mcpro.2021.100113 34139362 PMC8205261

[22] Zhao W Fogg DK Kaplan MJ . A novel image-based quantitative method for the characterization of NETosis. J Immunol Methods. (2015) 423:104–10. doi: 10.1016/j.jim.2015.04.027 PMC4522197 26003624

[23] Manda-Handzlik A Fiok K Cieloch A Heropolitanska-Pliszka E Demkow U . Convolutional neural networks–based image analysis for the detection and quantification of neutrophil extracellular traps. Cells. (2020) 9:508. doi: 10.3390/cells9020508 32102320 PMC7072771

[24] Bezanson J Edelman A Karpinski S Shah VB . Julia: A fresh approach to numerical computing. SIAM Rev. (2017) 59:65–98. doi: 10.1137/141000671

[25] Bradley D Roth G . Adaptive thresholding using the integral image. J Graph. Tools. (2007) 12:13–21. doi: 10.1080/2151237X.2007.10129236

[26] Otsu N . A threshold selection method from gray-level histograms. IEEE Trans Syst Man Cybern. (1979) 9:62–6. doi: 10.1109/tsmc.1979.4310076

